# Influence of high intensity ultrasound and calcium chelating agent on structural and physicochemical properties of casein micelles

**DOI:** 10.3389/fchem.2026.1754803

**Published:** 2026-02-13

**Authors:** Mufida Khalifa El Jabali, Ipek Altay Villebro, Naaman Francisco Nogueira Silva, Lucas Sales Queiroz, Heidi Olander Petersen, Joachim Andreas Sørensen, Federico Casanova, Aberham Hailu Feyissa

**Affiliations:** 1 Research Group for Food Production Engineering, National Food Institute, Technical University of Denmark, Søltofts Plads, Lyngby, Denmark; 2 Center of Natural Sciences, Federal University of São Carlos (UFSCar), São Carlos, Brazil; 3 Research Group for Analytical Food Chemistry, National Food Institute, Technical University of Denmark, Søltofts Plads, Lyngby, Denmark

**Keywords:** calcium chelation, casein functionality, casein structure, micellar casein concentrate, physicochemical properties

## Abstract

**Introduction:**

This study investigates the combined effect of ultrasonication time (UST) and disodium phosphate (DSP) - Na_2_HPO_4_, used as a calcium chelating agent, on the structural and physicochemical properties of casein micelles.

**Methods:**

Micellar casein concentrate (MCC) was prepared at 3 % (w/w) casein. DSP was added at concentrations of 0, 5, and 10 mM, followed by pH adjustment to 7.0. Ultrasonication was applied at 20 kHz and 550 W for 0, 10, and 20 min in pulsed mode (5 s ON/OFF) at 25 °C. Response Surface Methodology (RSM) was used to evaluate independent and combined effects of UST and DSP. The response variables included non-micellar calcium and magnesium, protein solubility index (PSI), hydrodynamic diameter (Dh), ζ-potential, surface hydrophobicity, viscosity and secondary structure.

**Results:**

Increasing both UST and DSP significantly increased non-micellar calcium concentration (from 238.4 to 327.2 mg/kg) and PSI (from 29.0 % to 44.1 %), while surface hydrophobicity decreased significantly (p < 0.0001). Dh decreased from 206.1 to 186.5 nm with increasing UST up to 10 min but increased with increasing DSP at all USTs (p < 0.0001). Increasing DSP led to a more negative ζ-potential, decreasing from –16.1 mV at 0 mM DSP to –20.4 mV at 10 mM DSP in the absence of sonication (p < 0.0001). DSP and UST showed no significant effect on viscosity. FTIR analysis indicated limited effects of the treatments on the secondary structure of caseins. Overall, the combination of ultrasound treatment and calcium chelation altered key physicochemical properties of micellar caseins, which may be explored in future studies to assess their impact on functional properties.

## 1 Introduction

Casein micelles are unique protein structures found in milk, which stabilized by a complex network of hydrophobic and electrostatic interactions among proteins, along with colloidal calcium phosphate (CCP), which acts as a cementing and neutralizing agent to maintain micellar integrity and stability (Holt and Horne 1996). Micellar casein concentrate (MCC) is a dairy ingredient produced via microfiltration (MF) of skim milk and contains high content of native casein micelles (Goulding et al., 2020). Commercially available in liquid (≥9% total protein), concentrate (≥22%), or powder (≥80%) forms, MCC has gained prominence over the past decade for its versatility in beverages, yogurt, and cheese products (Hammam et al., 2021). However, its limited solubility and emulsification properties often necessitate the use of calcium chelating agents (CCAs) to enhance micellar dissociation and improve solubility and heat stability (McCarthy et al., 2017). Another strategy adopted for improving the MCC technological properties was ultrasonication, which increased surface hydrophobicity of casein micelles, and allowed the production of micellar casein powder (MCP) containing bigger particles that improves its flowability (Song et al., 2021). High-intensity ultrasound (HIUS), typically operating at relatively low frequencies from 20 to 100 kHz, is an emerging technology in food processing that induces acoustic cavitation in liquid media, generating localized physical effects such as microjets, shear forces, shock waves, and turbulence, as well as chemical effects including the formation of reactive species (Silva and Chandrapala, 2020; Sun et al., 2014). In the literature, HIUS treatments are often described in terms of ultrasonic intensity (W/cm^2^), which represents the power delivered per unit area of the emitting probe and can vary widely depending on probe geometry and operating conditions (Silva and Chandrapala, 2020; Sun et al., 2014). HIUS can significantly alter the structure and functionality of dairy proteins, offering potential for improving protein solubility and stability (Carrillo-Lopez et al., 2021). Several studies were conducted on the potential improvements of the properties of MCC by ultrasound treatment (Lo et al., 2019; Madadlou, et al., 2009; Silva et al., 2018; Song et al., 2022; Song et al., 2021; Zhao et al., 2022). However, no previous work had studied the impact of adding CCA combined with HIUS on the properties of MCC. Therefore, this study aims to investigate the combined effect of HIUS and the addition of Na_2_HPO_4_ (disodium phosphate - DSP) – a typical food grade chelating salt used in the dairy industry - on the structural and physicochemical properties of MCC. By altering mineral equilibria as well as protein structure and protein–protein interactions through DSP addition and HIUS treatment, this approach influences the aggregation state and physicochemical properties of casein micelles. These modifications provide a basis for future studies aimed at evaluating how changes in physicochemical characteristics may influence functional properties, including gelling, emulsifying, foaming, thermal stability, and powder rehydration. The relevance of such effects is expected to depend on the specific requirements of the targeted food application and may extend beyond dairy systems.

## 2 Materials and methods

### 2.1 Material and chemicals

Micellar casein powder (MCP) (Promilk 852B Via Lacta, 5% total moisture, 81.5% total protein, 85% protein on dry matter, 92% casein, 8% total ash, 5% total moisture, 4% lactose, 2.4% calcium, and 1.5% fat) was kindly provided by Ingredia (Arras, France). MCP was stored in vacuumed food grade plastic bags at 4 °C for experimental use. Disodium hydrogen phosphate, sodium hydroxide, sodium-8-anilino-1-naphtalene sulfonate, and casein standard (Sigma Aldrich, Germany), sodium azide (Sigma Aldrich, China), L-aspartic acid standard (Sigma Aldrich, Japan), hydrochloric acid (Fisher Chemicals, Germany), nitric acid (SPS Science, France), wheat flour standard (Elementar Analysensysteme, GmbH, Germany) were used.

### 2.2 Reconstitution of micellar casein powder (MCP)

MCC was prepared using deionized water to obtain a suspension with a concentration of 3% w/w caseins. Sodium azide was added at 0.02% (w/w) to prevent microbial growth. The suspension was stirred at 500 rpm (IKA-WERKE, GMBH and CO. KG, Germany) at room temperature for 72 h (Lo et al., 2019; Madadlou, et al., 2009; Zhao et al., 2022).

### 2.3 Preparation of samples

DSP (Na_2_HPO_4_) was added to the suspension to obtain a final concentration of 0, 5, and 10 mM. The suspension was stirred (IKA-WERKE, GMBH and CO. KG, Germany) for 72 h at room temperature to ensure complete dissolution. The pH was adjusted to 7.0 ± 0.01 by adding 1 M NaOH or 1 M HCl (Huppertz et al., 2017; McCarthy et al., 2017; Sadat et al., 2017).

### 2.4 High intensity ultrasound (HIUS) treatment

100 mL from each sample was ultrasonicated in a double-jacketed glass vessel while temperature was kept below 25 °C. HIUS treatment was performed using a sonifier (SFX550, Branson, CT, United States), operating at a constant frequency of 20 kHz and power intensity of 550 W/cm^2^. The samples were ultrasonicated at an amplitude of 100% for 0, 10, and 20 min in pulsed mode (5 s ON and 5 s OFF) (Lo et al., 2019; Madadlou et al., 2009; Silva et al., 2018; Song et al., 2021; Zhao et al., 2022). The energy delivered to the samples were 0, 1,500, and 3000 J/mL, which are calculated by the following Equation 1:

$$AED=\frac{P\times t}{V}$$ (1)

Where *AED* is acoustic energy density (J/mL); *P* represents the output power (W); *t* is total ultrasonication time (s); and *V* is the volume of the sample (mL) (Lo et al., 2019; Monteiro et al., 2020; Rutkowska et al., 2017; Song et al., 2021; Song et al., 2022).

### 2.5 Non-micellar calcium and magnesium

To evaluate non-micellar calcium and magnesium, about 30 g of MCC suspensions were ultracentrifuged (L8-60 M Ultracentrifuge, Beckman, U.S.A.) at 20 °C and 23,000 rpm (RCF = 111,000 × g) for 90 min, and the supernatants were collected for measurements (Chandrapala et al., 2012). The contents of non-micellar calcium and magnesium were determined by inductively coupled plasma optical emission spectroscopy (Aglient 5800 ICP-OES, Santa Clara, CA, United States)following a microwave-assisted acid pressure digestion (Multiwave 7,000, Anton Paar, Graz, Austria) according to the principles of the European standard methods EN 13805:2014 and EN 16943:2017. Concentrated nitric acid (65% w/w; SPS, Science, Villebon sur Yvette, France) and ultra-purified water (18.2 mΩ cm at 21.5 °C; Milli-Q-Integral System, Merck, Darmstadt, Germany) were used for the digestion and dilution of the samples. Quantification was done by external linear calibration with internal standardization using yttrium to correct for instrumental drift. All standards were prepared from certified stock solutions (SPS, Science, Villebon sur Yvette, France) in 5% HNO_3_. Certified reference materials, DORM-5 (Fish protein, National Research Council Canada (NRCC), Ottawa, Ontario, Canada) were used for quality assurance of the analytical results. The values obtained for calcium and magnesium agreed with the certified values.

### 2.6 Protein solubility index (PSI)

PSI was measured in supernatants of the ultracentrifuged samples as described in Section 2.4. The protein content in the supernatants and the non-ultracentrifuged samples was measured with DUMAS method. In brief, about 1,000 mg of the samples were placed in crucibles and inserted into DUMAS equipment (DUMAS, rapid MAX N EXCEED, Elementar Analysensyteme GmbH, Germany). L-Aspartic acid, wheat and casein were used as standards. Total nitrogen content was determined and used for protein% calculation by using a conversion factor 6.38. The protein content expressed in % (w/w) of the sample (Chandrapala et al., 2012; Liu et al., 2014; Nascimento et al., 2023). PSI (%) was calculated as described in Equation 2:

$$\%\text{ PSI}=\frac{\text{protein}\;\text{content}\;\text{in}\;\text{supernatant}\;\left(\text{mg}\right)}{\text{protein}\;\text{content}\;\text{in}\;\text{non}-\text{ultracentrifuged}\;\text{MCC}\;\left(\text{mg}\right)}\times 100$$ (2)

### 2.7 Hydrodynamic diameter (Dh) and ζ-potential measurements

Dh and ζ-potential of the samples were measured by dynamic light scattering (DLS) method using Zetasizer Nano-ZS (Malvern Instruments, Worcestershire, United Kingdom) equipped with capillary cells. The refractive indices for the samples were set to 1.45 for the protein and 1.33 for the dispersant. The measurements were performed at room temperature in analytical triplicates for each sample (Lo et al., 2019; Nascimento et al., 2023; Yang et al., 2020; Zhao et al., 2022).

### 2.8 Surface hydrophobicity index (H_0_)

H_0_ of MCC suspensions was measured as described by Liu & Qin, (2021) with some modifications. A series of MCC concentrations (0.01, 0.05, 0.1, 0.2, 0.3, 0.4, and 0.5%) (w/v) were prepared using deionized water. Then, 50 µL of 10 mM sodium-8-anilino-1-naphtalene sulfonate (ANS) was added to 2 mL of the diluted samples and vortexed (SCIENTIFIC INDUSTRIES, INC, United States) for 10 s. The samples were incubated in the dark at room temperature for 30 min. After the incubation, 200 µL of each sample was transferred to a 96-well plate. The relative fluorescence intensity (RFI) of each dilution and corresponding blank (samples without ANS) was measured using a spectrophotometer (SPECTRAmax GEMINI, Molecular Devices, CA, United States) at excitation and emission wavelengths of 390 and 470 nm, respectively. The H_0_ value was determined as the slope of the fitted curve between RFI and protein concentration (Song et al., 2021, Song et al., 2022; Zhao et al., 2022).

### 2.9 Measurement of viscosity

Viscosity of MCC suspensions were measured using a stress-controlled rheometer (DHR-2, TA Instruments, Hullhorst, Germany) equipped with a DIN-standard concentric cylinder system. Measurements were taken about 25 mL of MCC suspensions at 20 °C. The shear rate ranged from 1 to 300 s^-1^. Viscosity was reported at the shear rate of 100 s^-1^. Ten equilibrium points were recorded for each data point (Silva et al., 2018; Song et al., 2021; Sun et al., 2014).

### 2.10 Analysis of secondary structure

The changes in the secondary structure of freeze-dried MCC were determined using a Fourier transform infrared spectroscopy (FTIR) (Perkin-Elmer Spectrum 100 spectrometer, Waltham, Massachusetts, United States). With a resolution of 4 cm^-1^ and 32 scans, the IR spectra of ultrasound treated and untreated MCC were obtained from 400–4,000 cm^-1^ (Sadat and Joye, 2020; Silva et al., 2018; Song et al., 2022; Yang et al., 2020).

### 2.11 Experimental design and statistical methods

Response Surface Methodology (RSM) with central composite design was employed to investigate the combined effect of the two independent variables; ultrasonication time (UST) and disodium phosphate concentration (DSP) on MCC suspension properties. The measured response variables included non-micellar calcium and magnesium, PSI, Dh, ζ-potential, H_0_, and viscosity. Three coded levels (−1, 0, +1) were assigned to each independent variable, corresponding to 0, 10, and 20 min for UST and 0, 5, and 10 mM for DSP, as shown in Table 1 (full experimental design and the values of all measured responses are detailed in Supplementary Table S2).

**TABLE 1:** Independent variables used in RSM with coded and un-coded levels.

Symbols	Independent variables	Coded levels
​	​	Low middle high
UST DSP	Ultrasound treatment time (min) Disodium phosphate salt concentration (mM)	−1 0 + 1
		0 10 20 0 5 10

A multiple regression model, the second order polynomial model (Equation 3) was fitted to experimental data obtained (Supplementary Table S2) to evaluate the effect of UST and DSP on all the responses:

$$Y=\beta_{0}+\beta_{1}X_{1}+\beta_{2}X_{2}+\beta_{1,1}X_{1}^{2}+\beta_{2,2}X_{2}^{2}+\beta_{1,2}X_{1}X_{2}+E$$ (3)

where *Y* represents the response variable*, X*_*1*_ and *X*_*2*_ are independent variables (UST and DSP, respectively), $\beta_{o},\beta_{1},\beta_{2},\beta_{1,1},\beta_{2,2},{\;\beta }_{1,2}$ are regression coefficients: *β*_*0*_ is the intercept; *β*_*1*_ and *β*_*2*_ are the linear effects; *β*_*1*_ *,*_*1*_ and *β*_*2*_ *,*_*2*_ are the quadratic effects; and *β*_*1*_ *,*_*2*_ is the interaction effect.

The significance of the regression terms in the response surface models was evaluated using analysis of variance (ANOVA). Overall model performance was assessed through the model p-value, lack-of-fit test, *R* ^2^, adjusted *R* ^2^, and RMSE and statistical significance was assessed based on p-values at p < 0.05, as reported in Supplementary Table S1. Experimental results are presented as mean ± standard deviation. The data was evaluated utilizing JMP Pro 15 software (SAS Institute Inc., Cary. NC).

## 3 Results and discussion

### 3.1 Non-micellar calcium and magnesium

**FIGURE 1:**
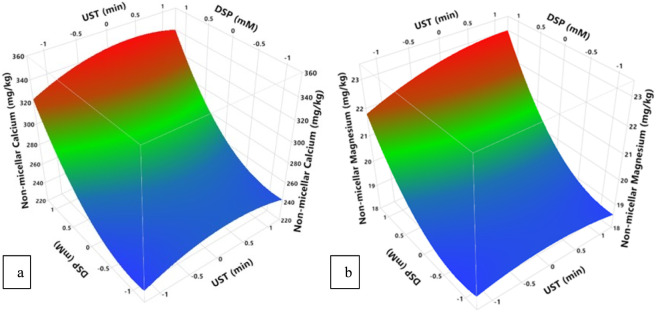
Effect of UST and DS on **(a)** non-micellar calcium, **(b)** non-micellar magnesium of treated MCC. UST and DSP axes are with the coded values (Table 1), where −1, 0, one represent actual values of 0, 10, and 20 min for UST and 0, 5, and 10 mM for DSP, respectively.

Casein-minerals interactions play a crucial role in formation and functional properties of casein micelles. The most important ions in relation to caseins are calcium and magnesium ions. They interact with negatively charged side functional groups such as carboxyl group of Glutamic and Aspartic amino acids, and the phosphate group of phosphoserines. These interactions depend on environmental conditions such as pH and ionic strength (Huppertz et al., 2017). Figure 1 demonstrates the concentration of non-micellar calcium and magnesium of treated MCC at different ultrasound time (UST) and DSP concentration. As shown by RSM analysis (Figure 1a; Supplementary Table S1) that non-micellar calcium significantly increases with increasing both UST (*p = 0.0009*) and DSP (*p < 0.0001*). As DSP directly affects mineral equilibria, the effect of DSP is higher than the effect of UST. Non-micellar calcium concentration significantly rose from 238.4 mg/kg at 0 min of UST and 0 mM of DSP to 321.8 mg/kg at 20 min of UST and 10 mM of DSP (Supplementary Table S2). For non-micellar magnesium concentration, it significantly increases with increasing DSP (*p = 0.0003*) and it is not affected by UST (*p = 0.2938*) (Figure 1b; Supplementary Table S1). In fact, magnesium is present at substantially lower concentrations than calcium (Supplementary Table S2), and under the experimental conditions used, variations in its concentration induced by ultrasonication time were small and not statistically significant. However, the non-micellar magnesium concentration increased significantly with increasing DSP concentration, independently of ultrasonication time (Supplementary Table S1; Figure 1a). The effect of ultrasound on calcium balance may be related to pressure effect caused by cavitation. High pressure probably induces disruption of ionic interactions between caseins and inorganic constituents, resulting in release of calcium from the micelles to the soluble phase. In milk protein systems, the removal of calcium ions has often been accompanied by protein structural changes, indicating the quaternary structure of casein was disrupted (Song et al., 2021). Casein micelles can be also disrupted through the solubilization of colloidal calcium phosphate, which can be achieved by the addition of calcium chelating agents (Huppertz et al., 2017). Song et al. (2021) investigated non-micellar calcium content for micellar casein concentrate 7.52% (w/w) sonicated at 20 kHz, out power 453 W, an amplitude 50% for 5, 10, 15, 30 and 60 min. They reported that there was a significant increase in non-micellar calcium from 320 to 384 mg/kg in long-time US treatments, especially at 30 and 60 min.

### 3.2 Protein solubility index (PSI)

**FIGURE 2:**
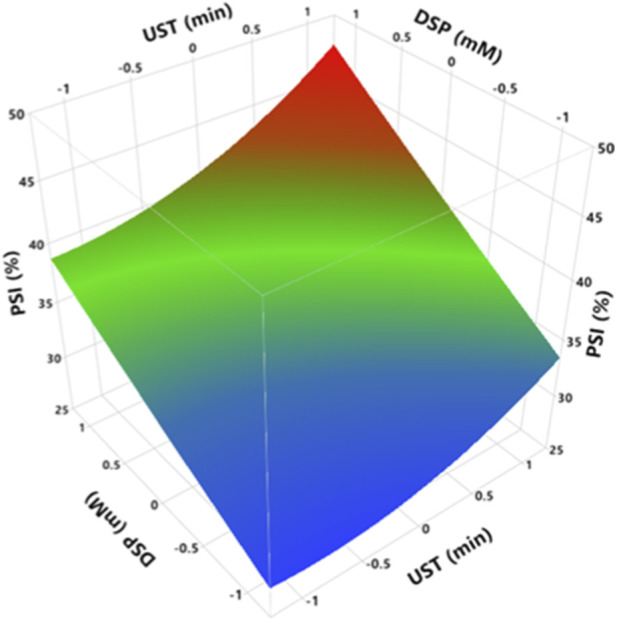
Effect of UST and DSP on PSI ofMCC. UST and DSP axes are with the coded values (Table 1), where −1, 0, 1 represent actual values of 0, 10, and 20 min for UST and 0, 5, and 10 mM for DSP, respectively.

The solubility of milk proteins plays an important role in most techno-functionalities in industrial applications, such as emulsifying, foaming, gelling, and viscosity functions. Milk protein solubility depends on particle size, protein surface hydrophobicity, and the degree of protein denaturation and aggregation (Shokri et al., 2022; Zhao et al., 2022). According to the RSM results, increasing both UST and DSP shows a significant increase (*p < 0.0001*) in the PSI values (Supplementary Table S1). UST has a parabolic impact on PSI, whereas DSP shows a linear trend (Figure 2). As displayed in Figure 2, PSI increased with increasing DSP, regardless of ultrasonication time. The highest increase in PSI was from 29.0% to 44.1%, which was recorded at 10 mM of DSP after 20 min of UST (Supplementary Table S2). A previous study reported that addition of 5, 10, 25, and 50 mM of sodium phosphate significantly increased the solubility of milk protein concentrate 10% (w/w) compared to the control (McCarthy et al., 2017). It is well known that the addition of calcium chelating agents to the milk system alters the calcium balance of the system, leading to a decline in the concentration of free calcium ions and a depletion of colloidal calcium phosphate (CCP) depending on the concentration and extent of chelation (McCarthy et al., 2017; Sadat et al., 2017). The depletion of CCP from casein micelles leads to their dissociation and the release of casein fractions into the continuous phase, altering the protein-mineral equilibria and increasing the repulsion between the negatively charged amino acids in the casein micelles. This results in an increase in hydration and voluminosity of the micelles (McCarthy et al., 2017; Sadat et al., 2017). Regardless of DSP, UST appeared to improve PSI from 29.0% at 0 min to 34.0% after 20 min of ultrasonication (Supplementary Table S2). The increasing effects of ultrasound on the solubility of milk proteins can be attributed to the decrease in particle size and the partial unfolding of casein structure consequent to the ultrasonic cavitation effect. This leads to exposing the hidden hydrophilic groups on the surface. These charged groups react with the surrounding water via electrostatic forces, leading to protein molecules dispersion, then increasing the solubility (Shokri et al., 2022). Han et al. (2020) obtained similar results on casein suspension (1.0 mg/mL), where the solubility of casein rose from about 65% to 72% by applying 300 W of ultrasound for 10 min. In addition, the solubility of MCC increased from 84% to 91% after HIUS pretreatment for 5 min at 20 kHz and a power density of 58 W/L (Zhang et al., 2018). However, Lo et al. (2019) recorded that ultrasonication at 20 kHz and 500 W did not affect the solubility of reconstituted sodium caseinate (4, 7, and 10% protein concentrations) at pH 6.7. PSI results are consistent with hydrodynamic diameter and hydrophobicity results (sections 3.3 and 3.5). PSI increased when hydrodynamic diameter and hydrophobicity decreased, as mentioned by Shokri et al. (2022) and Zhao et al. (2022) that milk protein solubility depends on particle size, protein surface hydrophobicity, and the degree of denaturation and aggregation.

### 3.3 Hydrodynamic diameter (Dh)

**FIGURE 3:**
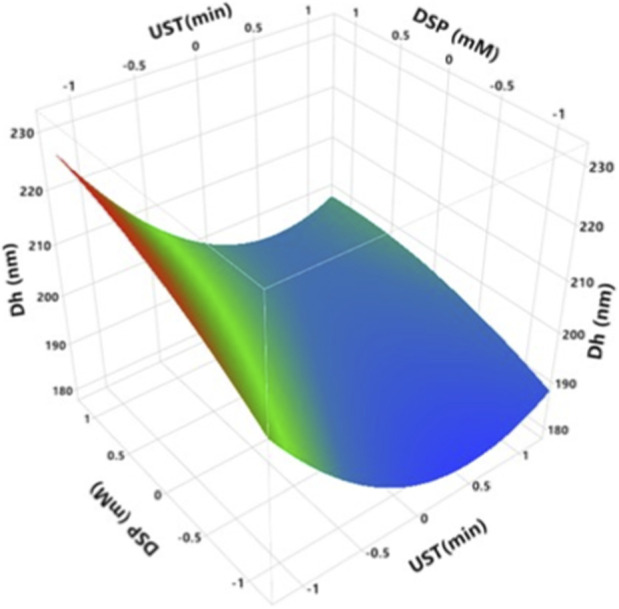
Effect of UST and DSP on Dh of treated MCC. UST and DSP axes are with the coded values (Table 1), where −1, 0, one represent actual values of 0, 10, and 20 min for UST and 0, 5, and 10 mM for DSP, respectively.

Particle size of milk proteins plays a crucial role in their techno-functional characteristics. Small particles have a higher surface area, which can increase solubility and enhance the emulsifying, foaming and gelling properties of milk proteins (Shokri et al., 2022). RSM results (Figure 3; Supplementary Table S1) shows that hydrodynamic diameter of treated MCC significantly decreases with increasing UST (*p < 0.0001*) and increases with increasing DSP (*p < 0.0001*). As shown in Supplementary Table S2, increasing DSP from 0 to 10 mM increased hydrodynamic diameter from 206.1 to 220.4 nm at 0 min. This result is in contrast with McCarthy et al. (2017) findings, who reported that 5, 10, 15, 25, and 50 mM of sodium phosphate did not significantly affect particle size of 10% w/w milk protein concentrates when compared with the control. A significant reduction in Dh occurred after 10 min of ultrasound treatment, where Dh decreased from 206.1 to 186.5 nm (Supplementary Table S2). Most of the studies have indicated that controlled or moderate ultrasound treatment induces a decrease in the particle size of milk proteins via structural disruption of the proteins. This is due to the disruptive effects of acoustic cavitation on hydrophobic and electrostatic interactions such as hydrogen bonds and van der Waals forces in protein three-dimensional structure, which can dissociate casein micelles into smaller particles (Shokri et al., 2022). In contrast, ultrasonication with relatively high intensities increases sulfhydryl group content that is buried within the interior of the protein due to partial unfolding of protein molecules which can react with themselves or be oxidized to form larger aggregates (Shokri et al., 2022). This result is consistent with the previous findings reported by Zhao et al. (2022) where the particle size of MCC (30 mg/mL) was reduced at pH 8 after 5 min of 20 kHz and 300 W ultrasonication.

### 3.4 ζ-potential

**FIGURE 4:**
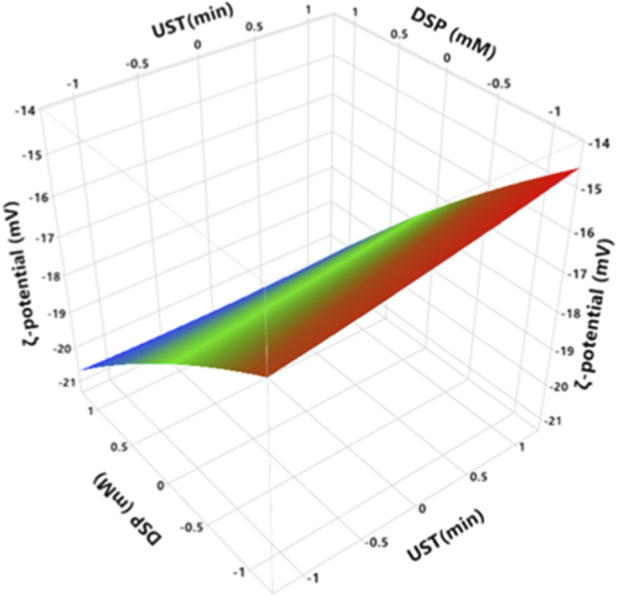
Effect of UST and DSP on ζ-potential of MCC. UST and DSP axes are with the coded values (Table 1), where −1, 0, and 1 represent actual values of 0, 10, and 20 min for UST and 0, 5, and 10 mM for DSP, respectively.

ζ-potential measures the net charge on the particle surface and the distribution of electric potential on the interface. This net charge is affected by pH, ionic force, and accumulation of surfactants agents (Cano-Sarmiento et al., 2018). Figure 4 shows the combined effect of UST and DSP on the surface net charge of MCC particles. RSM analysis (Figure 4; Supplementary Table S1) illustrates that ζ-potential significantly decreases with increasing DSP (*p < 0.0001*), i.e., casein micelles become more negatively charged, and significantly increases with increasing UST (*p = 0.0369*). As a matter of fact, the reduction of ζ-potential of casein micelles indicates more repulsion between the protein chains, and a lower tendency to aggregation (Zhao et al., 2022).

For non-ultrasonicated MCC samples, the values of ζ-potential decreased from −16.1 to −20.4 mV with an increase in DSP up to 10 mM (Supplementary Table S2). After 20 min of ultrasonication, the value of ζ-potential slightly but significantly increased from −16.1 to −14.9 mV at 10 mM of DSP (Supplementary Table S2). The results suggest structural and conformational changes of caseins, exposing more charged groups and enhancing electrostatic repulsion, which improves suspension stability (Cano-Sarmiento et al., 2018). Also, hydrodynamic diameter (section 3.3) was higher as the charge became more negative prior to sonication. Zhao et al. (2022) reported that the absolute value of ζ-potential of MCC (30 mg/mL) increased from 22.80 to 25.35 mV after ultrasound-assisted pH shifting treatment for 5 min at 20 kHz and 300 W, which is in line with the results presented herein. However, Lo et al. (2019) indicated that there were no changes in ζ-potential of sodium caseinate solution at pH 6.7 and 9.0 after sonication at 20 kHz and 500 W.

### 3.5 Hydrophobicity (Ho)

**FIGURE 5:**
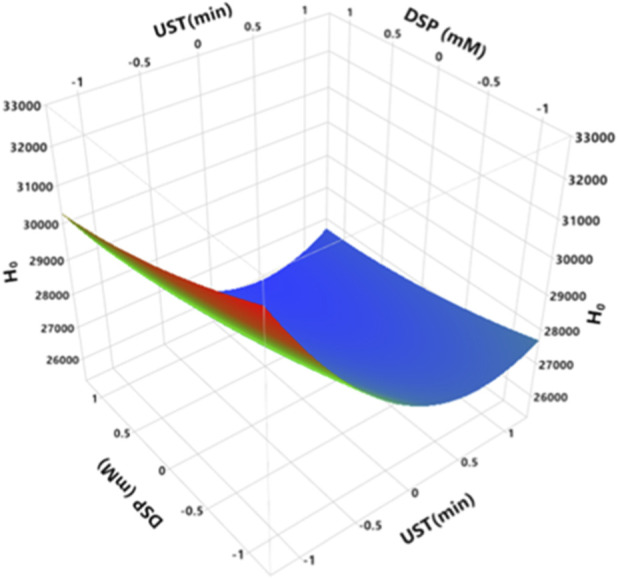
Effect of UST and DSP of H_0_ of treated MCC. UST and DSP axes are with the coded values (Table 1), where −1, 0, and one represent actual values of 0, 10, and 20 min for UST and 0, 5, and 10 mM for DSP, respectively.

Changes in surface hydrophobicity could be explained as a combined effect of the number and distribution of non-polar groups, and enlargement of hydrophobic amino acid residues that can improve water-protein interaction. At the same time, it can affect the stability of caseins (Han et al., 2020). Figure 5 displays H_0_ of treated MCC at different UST and DSP. Hydrophobicity index of MCC was significantly reduced by increasing both UST and DSP (*p < 0.0001*), as shown in Figure 5 and Supplementary Table S1.

The maximum reduction in H_0_ of treated MCC was after 10 min of ultrasonication, where H_0_ was reduced from 30,466.1 to 26,188.0, at 5 mM (Supplementary Table S2). The reduction in surface hydrophobicity of MCC may be due to the reduction in ANS-accessible hydrophobic sites, likely due to partial dissociation of casein micelles associated with calcium removal, which altered the exposure of hydrophobic domains. Zhao et al. (2022) investigated the effects of ultrasound combined with alkaline pH-shifting and reported an increase in the surface hydrophobicity of MCC at pH 8, 9, and 11, while decreases were observed at pH 10 and 12; notably, the highest surface hydrophobicity occurred at pH 11. These differences can be attributed to the use of extreme alkaline unfolding and refolding conditions in combination with ultrasound, whereas the present study employed DSP-mediated calcium chelation at neutral pH, which is expected to promote distinct structural responses and patterns of hydrophobic site accessibility. Han et al. (2020) studied the effects of high pressure and ultrasonic treatments on casein structure, and reported that ultrasounds affected the surface hydrophobicity index of casein solutions in a non-uniform manner, reaching a maximum value of 420.75 at 400 W of US treatment (the highest level of applied ultrasonic power).

### 3.6 Viscosity

Viscosity of protein solution relies on protein structure (size and shape), unfolding degree of protein molecule, and hydrophobicity of protein surface. Ultrasound has proved its role in modifying the flow behaviour (viscosity and consistency) of milk protein concentrates (Shokri et al., 2022). The effect of different UST and DSP on viscosity of treated MCC at shear rate 100 s^-1^ and 20 °C. RSM results (Supplementary Table S1) indicate that the model is not significant, indicating that both UST and DSP have no clear effect on the viscosity of treated MCC (UST and DSP). As shown in Supplementary Table S2, there is no difference in the viscosity of treated MCC with increasing DSP in all USTs. This result agrees with McCarthy et al. (2017), who found that adding 5, 10, 25, and 50 mM of sodium phosphate did not significantly affect viscosity profile of 10% (w/w) milk protein concentrate dispersions compared to the control at shear rate 100 s^-1^. Extending the ultrasound treatment to 20 min led to a reduction in viscosity, with no further differences observed among these longer treatment durations (Supplementary Table S2). It is reported that ultrasonication of milk protein rich in casein concentrates significantly reduces solution viscosity. Localized temperature, pressure, and shear force generated by acoustic cavitation disintegrate the large particles of casein micelles, which decreases viscosity of casein concentrates (Pegu and Arya, 2023). Similarly, Song et al., (2021) results showed that the viscosity of MCC samples 7.5% (w/w) displayed a decreasing trend with prolonging sonication time from 0 to 30 min at 20 kHz and 453 W at both 20 °C and 50 °C. In addition, Sun et al. (2014) reported a significant decrease in the apparent viscosity of milk protein concentrate solutions compared to the control after 5 min of ultrasonication at 20 kHz, 600 W, and 50% amplitude.

### 3.7 Secondary structure

**FIGURE 6:**
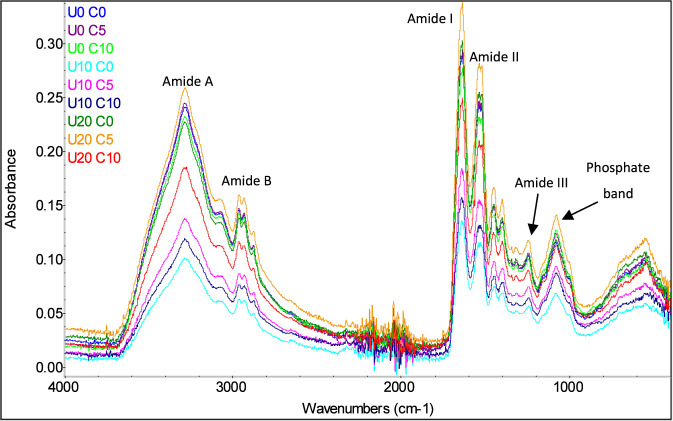
Peaks intensities of FTIR spectra of treated MCC at nine combined treatments of ultrasound time (U) for 0, 10, and 20 min and disodium phosphate concentration (C) at 0, 5, and 10 mM.

**FIGURE 7:**
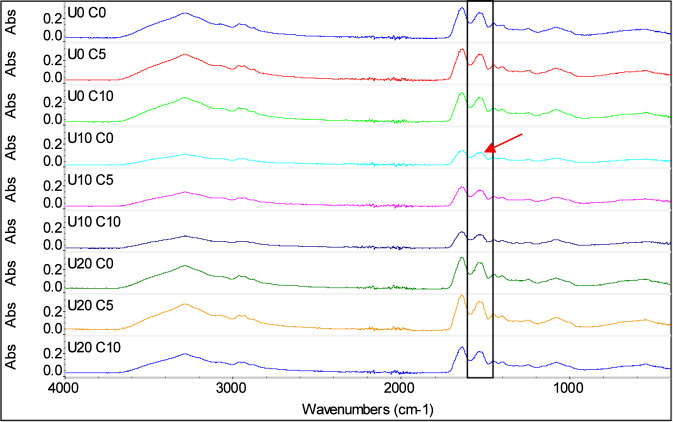
Peaks positions of FTIR spectra of treated MCC at nine combined treatments of ultrasound time (U) for 0, 10, and 20 min and disodium phosphate concentration (C) at 0, 5, and 10 mM.

FTIR spectra of MCC samples treated by nine combined treatments (0, 10, and 20 min of UST) and (0, 5, and 10 mM of DSP concentration) are shown in Figures 6, 7. FTIR is a handful technique that can provide important information regarding sample composition and structural modification. Herein, FTIR was employed to detect the amide bands of MCC, which are the primary spectral regions associated with proteins. The spectra (Figure 6) emphasize the main peaks and amide regions, reflecting key protein functional groups. The amide I band, observed in the 1700–1,600 cm^-1^ range, originates from C=O stretching vibrations in peptide bonds. Its frequency is also closely linked to the secondary structure of proteins. No shifts were noticed in the amide I region in the present work. Similarly, (He et al., 2022), reported that sodium caseinate samples did not show peak shifts at amide I region as well, when samples were treated by ultrasound. The amide II band, range of 1,550–1,500 cm^-1^, corresponds to in-plane N–H bending and C–N stretching vibrations. While it can be associated with protein secondary structure, it exhibits less sensitivity to protein conformational changes compared to the amide I band (Queiroz et al., 2021). However, as shown in Figure 7, amide II peak position has shifted from 1,532.04 cm^-1^ to 1,521.96 cm^-1^ when comparing control to treatment U10 C0.

High intensity ultrasound treatment has been extensively reported in the literature for its effect on protein secondary structure, depending on the chosen parameters, which suggests that the reported shift in amide II might be caused by physical effect of acoustic waves (Queiroz et al., 2025). In order to confirm how significant those changes were a deeper investigation using other techniques should be taken to evaluate the protein secondary structure in the Amide I and II regions. The results showed that peaks intensities of the full spectra were affected by all treatments.

## 4 Conclusion

Increasing ultrasound treatment time (UST) and disodium phosphate concentration (DSP) significantly altered the physicochemical properties of micellar casein concentrates. Protein solubility index (PSI) increased with both UST and DSP, while surface hydrophobicity (H_0_) decreased. Increasing UST led to a reduction in hydrodynamic diameter, consistent with changes in particle size, whereas increasing DSP up to 10 mM decreased the ζ-potential, indicating enhanced electrostatic stabilization of the micellar system. No effect on viscosity was observed. An inverse relationship between ζ-potential and hydrodynamic diameter was observed, with more negative ζ-potential values associated with larger particle sizes. FTIR analysis indicated that the applied treatments resulted only in minor modifications of spectral features, suggesting limited effects on the secondary structure of caseins under the studied conditions. Overall, the combination of ultrasound treatment and calcium chelation modified key physicochemical properties of micellar caseins. The implications of these modifications for functional properties—such as gelation, emulsification, foaming, and stability—remain to be evaluated, as higher or lower values of these properties may be advantageous or detrimental depending on the specific food application and desired product performance.

## Data Availability

The datasets presented in this study can be found in online repositories. The names of the repository/repositories and accession number(s) can be found in the article/Supplementary Material.
