# Supplementary material for: Influence of high intensity ultrasound and calcium chelating agent on structural and physicochemical properties of casein micelles
Source: Front Chem. 2026 Feb 13;14:1754803. doi: 10.3389/fchem.2026.1754803 (PMC12946099; doi:10.3389/fchem.2026.1754803)
Supplement: Supplementary file 1 [file Supplementaryfile1.docx]

Table S1. Effect of ultrasound treatment time (UST) and disodium phosphate salt (DSP) concentration on PSI, Dh, ζ-potential, H₀, Non-micellar calcium and Non-micellar magnesium from RSM.

| **Response** | | **PSI (%)** | **Dh (nm)** | **ζ-potential (mv)** | **H₀** | **Viscosity (mPa.s)** | **Non-micellar calcium (mg/kg)** | **Non-micellar magnesium (mg/kg)** |
| --- | --- | --- | --- | --- | --- | --- | --- | --- |
| **Coefficient** | **Intercept**  **p-value** | 34.5930  <0.0001*** | 194.5356  <0.0001*** | -17.7482  <0.0001*** | 26817.8490  <0.0001*** | 1.6617  <0.0001*** | 269.4100  <0.0001*** | 19.6139  <0.0001*** |
|  | **UST**  **p-value** | 2.9307  <0.0001*** | -10.6944  <0.0001*** | 0.2722  0.0369* | -1654.0350  <0.0001*** | -0.0412  0.0418* | 4.8634  0.0009*** | 0.2539  0.2938 |
|  | **DSP**  **p-value** | 5.050  <0.0001*** | 5.6852  <0.0001*** | -2.1778  <0.0001*** | -616.3186  <0.0001*** | 0.0337  0.0923 | 42.3656  <0.0001*** | 1.6631  0.0003*** |
|  | **UST*DSP**  **p-value** | 0.5137  0.1412 | -1.5056  0.2245 | -0.2000  0.1956 | 173.512  0.1782 | -0.0025  0.9178 | 1.2068  0.4138 | 0.1932  0.4895 |
|  | **UST*UST**  **p-value** | 1.3256  0.0098** | 9.4204  <0.0001*** | 0.0389  0.8559 | 1393.4782  <0.0001*** | 0.0407  0.2354 | -8.3213  0.0008*** | -0.1986  0.6222 |
|  | **DSP*DSP**  **p-value** | 0.1349  0.7810 | -1.996  0.2537 | -0.1667  0.4397 | 159.6034  0.3752 | -0.0012  0.9717 | 17.6200  <0.0001*** | 0.7450  0.1199 |
| **Model (p-value)** | | <0.0001*** | <0.0001*** | <0.0001*** | <0.0001*** | 0.1421 | <0.0001*** | <0.0031** |
| **R²** | | 0.9378 | 0.8984 | 0.9393 | 0.9458 | 0.2312 | 0.9876 | 0.9200 |
| **R²**  **adjusted** | | 0.9275 | 0.8742 | 0.9249 | 0.9329 | 0.1031 | 0.9780 | 0.9040 |
| **RMSE** | | 1.3597 | 4.1668 | 0.5183 | 431.5059 | 0.0950 | 4.6013 | 0.6093 |
| **Lack of Fit (p-value)** | | 0.0658 | 0.9084 | 0.0726 | <0.0001*** | 0.9999 | 0.3334 | 0.2907 |

Note: * p < 0.05, ** p < 0.01, *** p < 0.001 indicate statistical significance.

Table S2: Experimental design and value of measured responses: protein solubility index (PSI), hydrodynamic diameter (Dh), ζ-potential, surface hydrophobicity index (H_0_), viscosity, and non-micellar calcium and magnesium at different ultrasound treatment time (UST) and disodium phosphate salt (DSP) concentration.

| **UST (min)** | **DSP (mM)** | **PSI (%)** | **Dh (nm)** | **ζ-potential (mV)** | **H_0_** | **Viscosity (mPa.s)** | **Non-micellar calcium (mg/kg)** | **Non-micellar magnesium (mg/kg)** |
| --- | --- | --- | --- | --- | --- | --- | --- | --- |
| 0 (-1) | 0 (-1) | 29.0 ± 1.7 | 206.1 ± 5.2 | -16.1 ± 0.2 | 30466.1 ±11.1 | 1.7 ± 0.1^a^ | 236.3 ± 5.7 | 18.6 ± 2.1 |
| 0 (-1) | 5 (0) | 33.2 ± 0.6 | 213.4 ± 7.3 | -17.7 ± 0.2 | 30216.2 ± 57.0 | 1.7 ± 0.1^a^ | 252.0 ± 6.0 | 18.9 ± 2.2 |
| 0 (-1) | 10 (1) | 37.1 ± 2.0 | 220.4 ± 8.5 | -20.4 ± 0.4 | 29233.0 ± 45.9 | 1.8 ± 0.1^a^ | 312.2 ± 7.5 | 21.5 ± 2.5 |
| 10 (0) | 0 (-1) | 28.7 ± 0.3 | 186.5 ± 2.1 | -16.1 ± 0.5 | 28255.3 ± 110.4 | 1.6 ± 0.1^a^ | 243.3 ± 5.8 | 19.3 ± 2.2 |
| 10 (0) | 5 (0) | 34.5 ± 0.9 | 194.9 ±0.9 | -17.8 ± 0.3 | 26188.0 ± 181.8 | 1.7 ± 0.1^a^ | 265.5± 6.4 | 19.2 ± 2.2 |
| 10 (0) | 10 (1) | 40.8 ± 1.3 | 198.2 ± 2.9 | -19.7 ± 0.7 | 26329.5 ± 54.6 | 1.7 ± 0.1^a^ | 322.4 ± 7.7 | 22.0 ± 2.5 |
| 20 (1) | 0 (-1) | 34.0 ± 1.3 | 186.7 ± 2.3 | -14.9 ± 0.5 | 26846.9 ± 92.9 | 1.6 ± 0.1^a^ | 244.8 ± 5.9 | 18.2 ± 2.1 |
| 20 (1) | 5 (0) | 38.8 ± 1.1 | 194.1 ±1.6 | -17.7 ± 0.2 | 26836.3 ± 75.2 | 1.7 ± 0.1^a^ | 267.6 ± 6.4 | 20.5 ± 2.3 |
| 20 (1) | 10 (1) | 44.1 ± 1.2 | 194.9 ±1.6 | -20.1 ± 0.7 | 26307.9 ± 44.6 | 1.7 ± 0.1 | 323.8 ± 7.8 | 21.8 ± 2.5 |

Note: In the 1^st^ (USP) and 2^nd^ (DSP) columns, the coded value of the test parameters is in parentheses, and the real (un-coded) values are outside the parentheses. The coded values -1, 0, and 1 represent low, middle, and high, respectively (Table 1). Data presented as mean ± standard deviation.
